# Hyperspectral characterization of natural lighting environments

**DOI:** 10.1016/bs.pbr.2022.04.008

**Published:** 2022-01-01

**Authors:** Takuma Morimoto

**Affiliations:** 1 Department of Experimental Psychology, University of Oxford, Oxford, UK; 2 Department of Experimental Psychology, Justus-Liebig-Universität Gießen, Gießen, Germany

**Keywords:** spectral measurement, natural lighting environment, hyperspectral imaging, hyperspectral illumination map, computer graphics

## Abstract

Lights are primary drivers of some crucial biological functions including vision and regulation of circadian rhythm. To understand the light exposure pattern that we experience in a daily life, many past studies measured the spectral composition of natural daylight and artificial lighting. The aim of this book chapter is to introduce a novel method to characterize directional spectral variation in natural lighting environments. An omnidirectional hyperspectral illumination map stores the spectra of lights coming from every direction towards a single point in a scene. Such illumination maps allow us to simulate a spatial light exposure pattern that reaches our eyes, providing useful resources to research areas such as chronobiology, vision science and any other fields which benefit from knowledge about the spectral nature of visual lighting environments.

## 1 Introduction

A vast majority of living organisms, including humans, use light to generate vision and guide themselves to effectively interact with the surrounding world. For a long time, the first stage of the human visual system was thought to be mediated by four types of photoreceptors in our retinae: rod cells and three classes of cone cells which have spectral sensitivities that peak at different wavelengths. However, around 20 years ago a subset of ganglion cells was found to contain a photopigment called melanopsin (Provencio et al, 2000), which is capable of directly sensing light. This fifth photoreceptor class is now widely recognized as intrinsically photosensitive retinal ganglion cells (ipRGCs). Since their discovery, their physiological properties have been carefully characterized (Dacey et al., 2005). Furthermore, tireless efforts have identified that ipRGCs contribute to vital biological functions such as regulating circadian rhythms (Wright & Lack, 2001), melatonin suppression (Prayag et al., 2019), signaling an acute alertness (Cajochen, 2007; Souman et al. 2018), and controlling pupil dilation (Gamlin et al., 2007). In addition to these non-visual functions, some studies reported a contribution of ipRGCs to visual functions such as perception of brightness of a light (Brown et al, 2012, Delawyer et al, 2020).

Given that lights form some vital biological functions, it is of a natural interest to characterize the spectral properties of the natural lighting environment. However, it is challenging because the lighting environment is complex and dynamic. The self-rotation of the Earth greatly changes the color and intensity of the daylight spectra that reaches the ground within a day. Over a year the seasonal change creates a gradual variation in the color distribution of natural environments, which then alters light reflected to our eyes from the environment (Webster et al., 2007). Despite these challenges many statistical regularities were identified. One widely known work is an analysis of 622 daylight spectra (Judd et al, 1964) collaboratively measured in the UK (Henderson & Hodgkiss, 1963), Canada (Nayatani & Wyszecki, 1963) and the US (Condit & Grum, 1964). It was found that the color of natural daylight mainly changes along the blue-yellow color direction. They further applied principal component analysis to the spectra and showed that it is possible to express a spectrum of typical daylights based on a linear model that uses only three basic functions, showing a high statistical regularity in natural daylights. This work is now widely recognized as the Commission internationale de l’éclairage (CIE) daylight model. Subsequent studies found that this trend generally holds in other countries (Das & Sastri, 1965; Dixon, 1978; Nayatani et al. 1967; Sastri, & Das 1968;Sastri & Manamohanan 1971; Winch et al. 1966). Moreover, some studies performed a large-scale measurement of outdoor illuminations (Hernández-Andrés et al., 2001) and indoor lightings (Houser et al, 2013). Other studies characterized the atmospheric condition such as how cloud coverage, the amount of water vapor in the air, or air pollution can alter daylight spectra (Tian et al, 2016; Spitschan et al. 2016). These measured datasets undoubtedly advanced our understanding of the mechanisms underlying visual functions. For example, to stably derive information about a surface color from an object, our visual system might effectively use the constraint that the illumination in a natural environment is likely to change along the blue-white-yellow axis (Maloney & Wandell, 1986).

Yet, past studies mostly measured the spectral irradiance of daylight using a spectrophotometer that was placed at a space widely open to the sky such as the roof top of a building. This method summarizes lights coming towards a single point from wide angles by a single spectrum, and thus the angular resolution is lost. While this single-spectrum measurement is valuable, it rarely accounts for the practical light exposure pattern we experience in daily life. In the real-world daylight does not always directly reach our eyes. Instead, light rays emitted from a light source are reflected and transmitted by objects that exist in a scene. Thus, both direct emission and secondary (or higher order) reflection contribute to natural lighting, meaning understanding the natural lighting environment requires a measurement to capture directional spectral variation.

In the computer graphics field, this directional dependency of the lighting environment is approximated using a tool called *environmental illumination map* (often also referred as lightprobe). It is a two-dimensional image where each pixel stores information about the incident light coming from a particular direction towards a single point in a scene. This tool has shown itself to be efficient in creating compelling imagery. There are several online databases; however, current publicly available lightprobes were created using standard cameras with three spectral channels (namely, Red, Green and Blue) and thus at each pixel spectral information is lost. For many applications RGB datasets are sufficient; however, for a field that concerns the wavelength-dependent effect of lighting spectral information is essential. Thus, in a previous study (Morimoto et al, 2019), my colleagues and I created a hyperspectral environmental illumination map using a hyperspectral imaging technique. The goal of this chapter is to introduce the methodology to capture such an illumination map which stores spectral information of all lights converging at a single point in a scene of interest. At the end of chapter potential applications of the measured datasets are also discussed.

## 2 Method

A methodology to create an environmental illumination map was first introduced by Debevec (2005), and in our measurement an RGB standard camera was replaced with a hyperspectral camera. There are several ways to measure a hyperspectral illumination map. One relatively simple way would be to photograph a perfectly mirrored sphere at the test location in the scene using a hyperspectral camera. Photographing a mirror sphere allows us to capture the light reflected from the sphere, which in turn give us the measure of the light rays hitting the sphere from a wide range of incident angles. An alternative method would be to use a fish-eye lens which can capture the light from within a half hemisphere in front of the camera with a single shot. Thus, in theory only two images can cover the whole scene. However, this method may be more susceptible to chromatic aberration when a hyperspectral camera is used. Another method would be to capture many images by rotating the camera and to later stitch the images together to cover the whole scene. Each method has different advantages and disadvantages, and thus ultimately readers should decide what methodology suits their purpose. For example, if a single data acquisition takes too long, the third approach of taking many images may not be suitable because the lighting environment may change during the measurement. In this book chapter the first method using a mirror sphere is introduced, but most of these measurement tips can be useful for other methods as well.

### 2.1 Capturing images of a mirror sphere

First of all, we need to choose a location of interest; there are several tips here. First, the ground at the location should be flat to minimize the misalignment of the imaging system. Second, it is important to make sure that lighting environments stay constant as much as possible during the measurement because hyperspectral measurement can take a long time, although exact acquisition time depends on the type of the imaging system and overall light level in the environment. For example, scenes including foliage may be avoided on a windy day. Also, no one should walk into a scene during the measurement. Although the change of the sun’s position during the measurement is inevitable, it may be worth choosing a time when the sun’s position is reasonably stable as opposed to around sunrise or sunset. The dynamic range of the scene is another challenge. In our previous study we performed all outside measurements in shadow to avoid direct sunlight. However, readers may instead choose to capture a mirror sphere with different exposure times and synthesize the images later to create a high dynamic range image. Another practical challenge is to secure the power supply for the imaging system and a control computer (if required), and thus a location close to a building may be a convenient choice.

**Figure 1:**
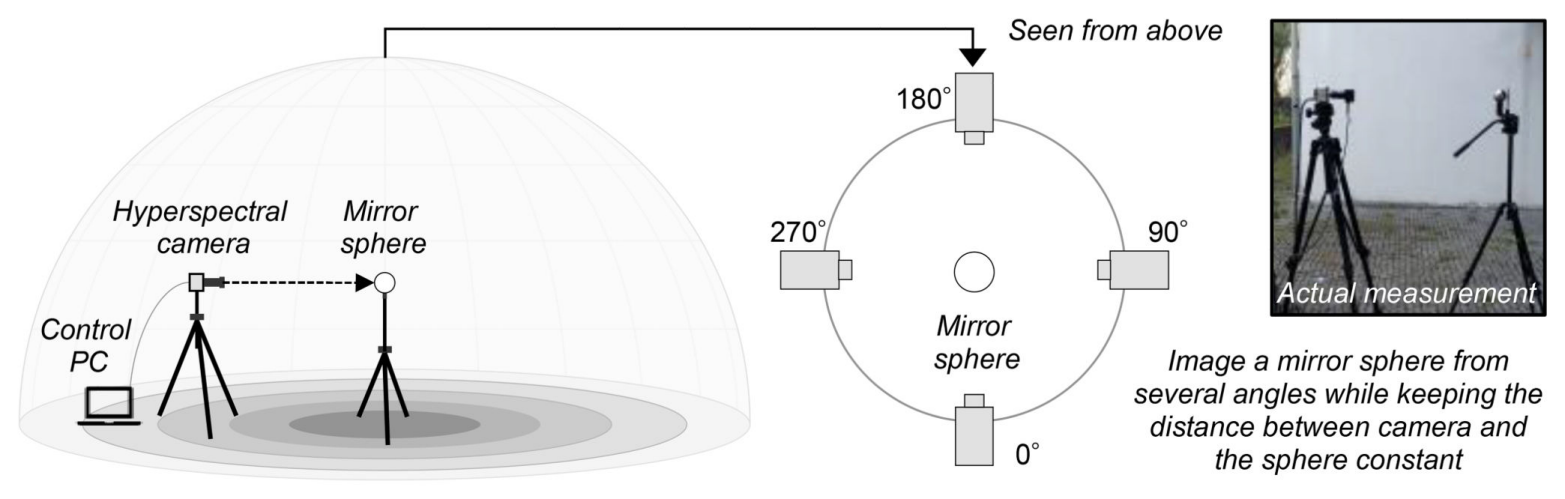
Imaging a mirror sphere using a hyperspectral camera. The image should be taken from several angles and stitched together in the subsequent image processing. The righthand picture shows the actual measurement used in a previous study.

Once the target location is selected, a hyperspectral imaging system and a mirror sphere should be set up as shown in Figure 1. The mirror sphere can be placed on top of an optical support attached to a tripod. The height of the sphere should be chosen according to the purpose of the measurement. For example, one may wish to measure light at the height of human eyes. Note that illumination maps generally assume that incident lights hit the sphere from the point of infinity. Meeting this assumption completely is practically challenging, but it is important to make sure that there is nothing immediately nearby the mirror sphere. If available, the zoom of the lens should be maximized to allow the largest physical distance between the imaging system and the mirror sphere. This in turn minimizes the size of the image of the imaging system reflected in the mirror sphere. Then, the distance between the imaging system and the mirror sphere can be determined such that the image of the mirror sphere fits just within upper and lower limits of the overall image.

A mirrored sphere reflects nearly the entire environment surrounding it, which is a much wider range than the hemisphere facing toward the imaging system. However, there are at least three issues in trying to create a lightprobe with a single image. First, towards the edge of mirror sphere the reflected image of the surrounding environment becomes highly distorted. This means that a wider angular region in the surrounding environment is mapped onto a single pixel, leading to low angular resolution around the edge. Second, the region immediately behind the sphere is not reflected in the sphere. Third, the imaging system itself is reflected on the mirror sphere, which thus blocks the lights coming from behind the imaging system towards the sphere. Thus, it is preferable to take several images of the mirrored sphere from different camera positions. Each sphere image can be processed independently and several angles can be stitched together in a later image processing stage.

To provide a concrete example, I will introduce some specifications of the hyperspectral imaging system used in the previous study (Morimoto et al, 2019). The imaging system is based on a Peltier-cooled digital camera (C4742-95-12ER, Hamamatsu Photonics K.K., Hamamatsu, Japan). The camera has a spatial resolution of 1344 × 1024 pixels and spectral resolution of 33 channels from 400 nm to 720 nm with 10 nm steps, achieved by a tunable liquid-crystal filter (VariSpec, model VS-VIS2-10- HC-35-SQ, Cambridge Research & Instrumentation, Inc., MA). The imaging system was a wavelength scanning type, meaning that one image of a specific wavelength band was captured per acquisition and measurement was repeated until all wavelength channels were recorded. We adjusted the focal length before measurement. The aperture was set to F16. The intensity resolution was 12-bits per wavelength channel. Before each acquisition the camera needs to be aligned horizontally using a spirit level. The exposure time for each wavelength acquisition was determined before the measurement so that enough light can be captured without complete saturation, and the set exposure times were kept constant for all angles. At the end of measurement, a dark-field image should be acquired with the hyperspectral camera covered with the lens cap, using the same exposure time. This image is used in post-hoc calibration process.

### 2.2 Image processing

**Figure 2:**
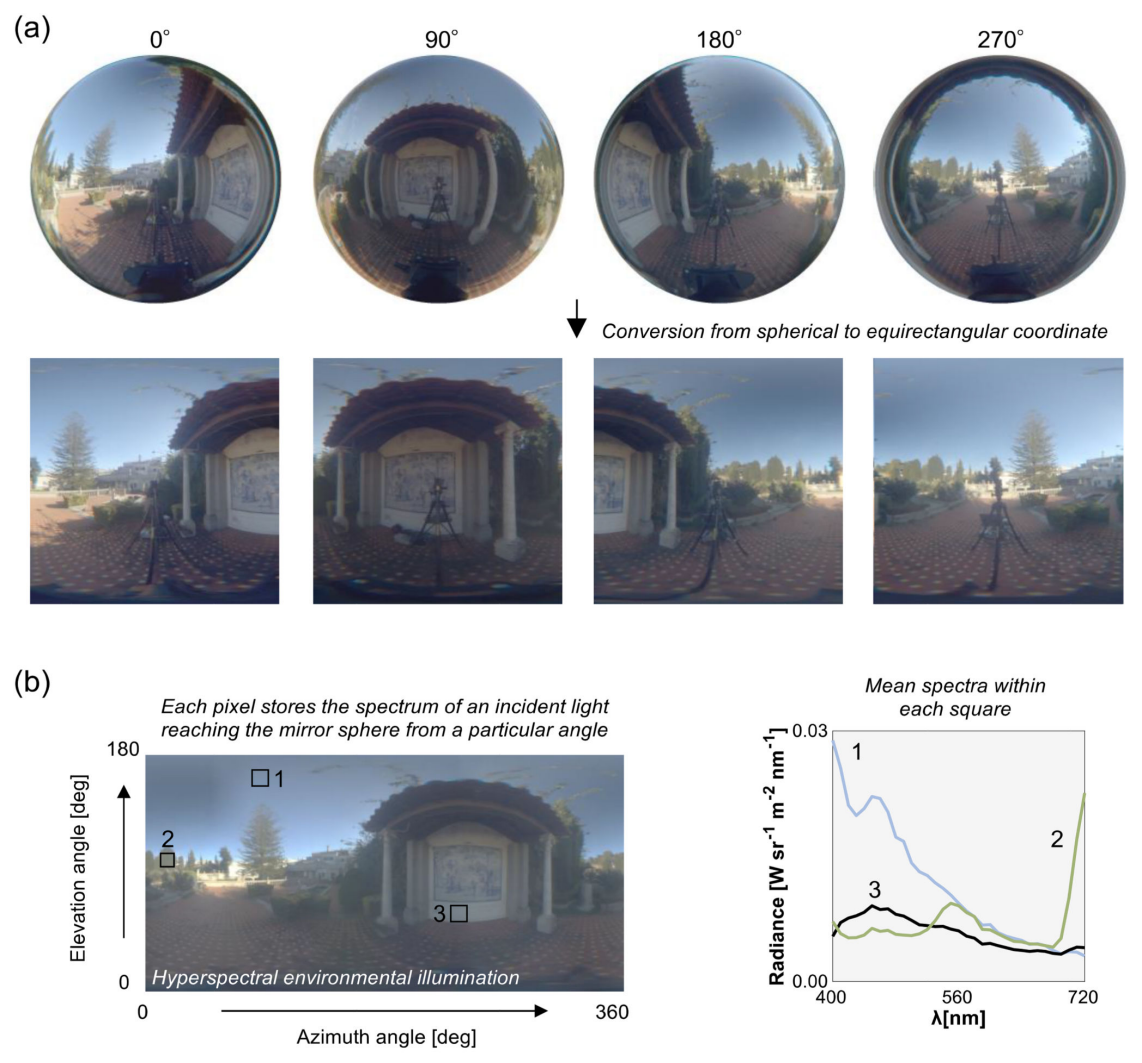
An image processing method to create a hyperspectral environmental illumination map. (a) Example images of a mirror sphere and each mirror sphere is “unwrapped” to be converted to equirectangular coordinate. Note that the horizontal edges of these unwrapped images were cropped because these regions have low angular resolution and also chromatic aberration. (b) Generated hyperspectral environmental illumination. The right-hand subpanel indicates spatial mean spectra across all pixels in each numbered square. It is shown that there is a large directional spectral variation within a scene.

This subsection introduces a brief overview of the image processing pipeline but the detailed procedure is provided in the previous study (Morimoto et al, 2019). The overall goal of this image processing is to produce an omnidirectional environmental illumination map from a set of mirror sphere images. The approach here is to process images taken at different camera positions independently first, and lastly stitch the processed images together. The upper part of Figure 2 (a) shows example sphere images taken at four different camera angles.

First, the average amount of dark noise in the imaging system needs to be subtracted from all pixels in a sphere image, using the measured dark-field image. Subsequently, spatial in-homogeneity of the imaging system due to off-axis vignetting (i.e. darkening toward the edge of the image) should be calibrated.

At this stage, the sphere image has arbitrary units (e.g. integers from 0 to 4095 for 12 bit-depth). However, it is desired to calibrate the image so that each pixel stores a spectrum with an absolute unit (e.g. spectral radiance [W sr^-1^ m^-2^ nm^-1^]). One approach is to place a matte reference plate with a reasonably flat spectral reflectance next to the sphere and include this plate in the image when taking a measurement. Then, a spectrum reflected back from this plate should be measured immediately after each acquisition using a spectroradiometer. This spectrum serves as a ground-truth and can be used to equate the spectrum at the reference plate recorded by the hyperspectral camera to this ground-truth spectrum independently measured by the spectroradiometer.

Then, any saturated pixels should be removed from the image. The wavelength-dependent specular reflection function of the mirror sphere should be measured and subtracted from the image. For the estimation of specular reflectance of the sphere, a specialized spectrophotometer is normally used. Then, if the noise level at each pixel is too high, the spectra may need to be smoothed to reduce the noise level.

After these corrections, each sphere image may be converted to equirectangular coordinates. This is a transformation in which the horizontal coordinate represents the azimuth and the vertical coordinate indicates the elevation. The elevation zero corresponds to the height of the camera. Note that this conversion is an optional choice and one may choose to work in spherical coordinates.

If the level of chromatic aberration is high, this may need to be corrected. In a method using a mirror sphere, the chromatic aberration is mainly seen at the edge of the sphere. Thus, the chromatic aberration is a less of an issue as these regions around the edge are not used in the final product. Nevertheless, one may choose to perform image rectification to reduce the influence of chromatic aberration.

Finally, remove the reflection of the imaging system in each sphere and fill in the blank pixels using corresponding region captured at another angle. Then, the sphere images should be stitched together, for example based on a stitching algorithm, to generate a 4π steradian omnidirectional illumination map as shown in Figure 2 (b). The plot on the right-hand side shows three example spectra recorded in three different regions in the illuminant map, showing that there are substantial directional spectral variations in this environment.

This section introduced a basic procedure to process collected sphere images; however, note that additional constraints may be introduced depending on the measurement setup, and it is important to develop an appropriate image processing pipeline that is tuned to individual datasets.

## 3 Application

Analyzing the illumination map itself (as briefly shown in the spectral plot of Figure 2 (b)) can be a powerful way to characterize the statistical variability in natural lighting environments. Nevertheless, it is worth noting that these illumination maps can be directly imported to state-of-the-art computer graphics software (e.g. *Mitsuba 2* developed by Nimier-David et al. (2019)) to generate a hyperspectral image of an arbitrary object embedded in the environment.

**Figure 3:**
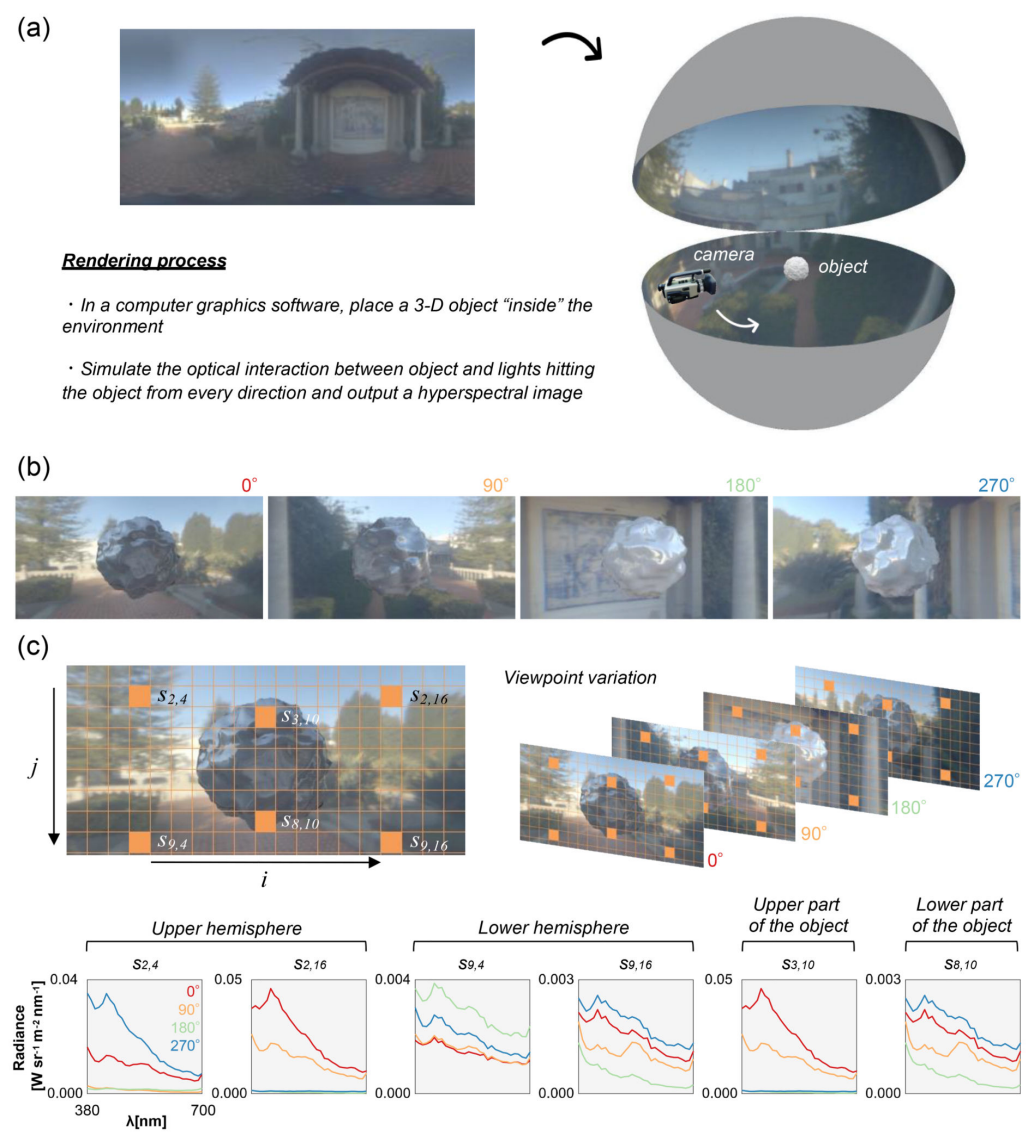
A rendering process to synthesize the object inside the measured lighting environment. (a) In computer graphics software a three-dimensional object is placed inside the environmental illumination and optical simulation of light-material interaction is performed to generate an image of the object embedded in the environment. (b) Some example images of a rendered object from different camera angles. (c) Drawing a grid on each image and spectral plots to show how mean spectra in six selected grid cells change as the camera position changes.

Figure 3 (a) shows a process to generate such a hyperspectral image. We first put a three-dimensional object of interest inside the environment as shown in right-hand side of panel (a). For illustrative purposes an object is placed in a sphere with a certain radius. However, it is assumed that incident lights reach the center object from the point of infinity. Thus, it should be thought that this object is placed in an infinitely large sphere. Then rendering software optically simulates the interplay between lights hitting materials and surface properties of material to produce the spatial light pattern reflected from the objects to the camera.

Figure 3 (b) shows the example images of a bumpy object rendered under the environment (using the rendering software *Mitsuba 0.6*). This bumpy object was set to have both a diffuse and specular reflectance of 50% across all wavelengths. Then, hyperspectral rendering (31 channels; 400 - 700 nm with 10 nm steps) was performed from four different camera angles (0°, 90°, 180° and 270°) while keeping the distance between camera and the bumpy object constant. Note that the raw output images were hyperspectral images, but they were converted to sRGB images for display presentation. It is important to note that these images can be thought as an approximation of the light exposure pattern projected onto our retina (see Webler et al. (2019) in which the idea of “spectral diet” was introduced). Thus, if we draw a grid, as seen in panel (c), we can estimate how much photoreceptors that are in charge of each spatial grid are activated by these images.

The bottom plot shows how mean spectra in six selected grid cells (labeled *s_2,4_, s_9,4_, s_3,10_, s_8,10_, s_2,16_*, and *s_9,16_*) change over camera positions (i.e. viewing angles). Here grid cells *s_2,4_* and *s_2,16_* correspond to small regions in the upper hemisphere of the scene. The spectral plot shows that spectra in each cell grid change dramatically as we change the viewpoint. Next if we look at spectra in s_9,4_ and s_9,16_, which are presumably dominated by secondary reflection from the ground and other objects in the scene, we again see spectral change as a function of viewpoint, but overall light levels are nearly one digit lower than spectra in the upper hemisphere (*s_2,4_* and *s_2,16_*). A similar trend holds between upper and lower part of the object (*s_3,10_* and *s_8,10_*). These demonstrate that retinal light exposure patterns can be complex over space and time. However, they also exhibit a curious systematic regularity.

In general, measured datasets can be useful for studies that benefits from the characterization of natural lighting environments. For example, it seems that the upper and lower part of our retina are on average adapted to drastically different light levels. How does the circadian system deal with such differences to function as a whole system? Also, since the spectral information is recorded, it is also possible to simulate the excitation of photoreceptors when seen by other species. Such applications could be useful for studies in visual ecology, to help understand the light exposure pattern experienced by living organisms other than humans.

In vision science understanding mechanisms that underpin stable perception of material properties is a fundamental domain. For example, humans can easily identify a range of visual properties such as metal, cloth, or plastic. Moreover, we are skilled at inferring their associated physical properties, such as light/heavy, soft/hard, rough/smooth, or even their states, such as wet/dry, clean/dirty, solid/melted. We have a good understanding of how we perceive such complex properties under simple lighting environment where a single light source illuminates the material, but mechanisms under complex lighting environments are still poorly understood. The measured dataset provides a valuable tool in tests of material perception as it allows production of physically accurate and realistic materials which can be used as experimental stimuli.

These measurements are also relevant to industry. For the display and lighting industry understanding the availability of color signals in natural environments and how humans sense these signals can inform ergonomic display and lighting design. In the computer graphics industry these data could be used to develop compelling imagery of objects placed under real-world lighting conditions. Moreover, industries, such as dye or paint, can use the data to simulate the visual appearance of surface treatments under different lighting environments.

Finally, there are recent efforts to measure environmental illumination. Shiwen et al. (2021) developed a portable imaging system and using the system they measured a hyperspectral lightprobe in a forest environment. Nilsson & Smolka (2021) also carefully characterized a lighting environment in a forest, though a standard RGB camera was used. Hirai et al. (2019) used a six-band multispectral camera to capture a high-spatial resolution illumination map. Moreover some exciting future challenges are foreseen, such as how time-lapse measurement of lightprobes characterizes the extent to which a set of light reaching a certain point in a scene varies over time and the change of directional spectral variation associated with temporal components in the environment (e.g. sun elevation). Similarly, given that surface reflectance properties of scene materials could largely change across seasons (e.g. forage of plants and leaves), it would be desirable to characterize the lighting environments across seasons.

This chapter introduced a method to characterize an overlooked aspect of the natural lighting environment. The amount of datasets collected is currently limited, and it is important future studies expand them. Nevertheless, hyperspectral illumination maps provide useful resources to research fields that are concerned about the nature of visual lighting environments.

## 4 Conclusion

A methodology to characterize the novel aspect of natural lighting environment was introduced. Measured data provides an image-based representation of a natural lighting environment, which stores a rich number of spectra that reaches a single point in a scene from every incident angle. By feeding the measured data into computer graphics software it is possible to synthesize an arbitrary object in the lighting environment. Measured datasets can be useful to directly characterize the light exposure pattern that circadian and visual systems experience in daily life.
